# Supplementary material for: Knowledge of Human Mpox (Monkeypox) and Attitude towards Mpox Vaccination among Male Sex Workers in China: A Cross-Sectional Study
Source: Vaccines (Basel). 2023 Jan 28;11(2):285. doi: 10.3390/vaccines11020285 (PMC9966706; doi:10.3390/vaccines11020285)
Supplement: Supplementary file 1 [file vaccines-11-00285-s001.zip › vaccines-2144094-supplematary material-Tables.pdf]

Table S1. Knowledge of mpox and mpox vaccination among male sex workers in China

No.	Knowledge of mpox	Answers
1	Mpox spreads via person-to-person close contact	**Agree**<br>Disagree
2	Reinfection of mpox can happen	**Agree**<br>Disagree
3	Most mpox cases recover within weeks	**Agree**<br>Disagree
4	Most mpox cases are HIV-positive	**Agree**<br>Disagree
5	HIV-infected and HIV-uninfected mpox cases have similar clinical signs	**Agree**<br>Disagree
6	HIV ART* may prevent mpox	**Agree**<br>Disagree
7	Mpox infection has sequelae	**Agree**<br>Disagree
8	MPXV is detectable in semen	**Agree**<br>Disagree
9	MPXV is unlikely to have genetic mutations	**Agree**<br>Disagree
10	Smallpox vaccine exists cross-protection against mpox	**Agree**<br>Disagree
11	Smallpox vaccine has intensive protection against mpox	**Agree**<br>Disagree
12	Mpox vaccination has side effects	**Agree**<br>Disagree
13	Mpox vaccine is not suitable for pregnant women, people who have heart problems and skin conditions	**Agree**<br>Disagree
14	Replicating mpox live attenuated vaccine is not suitable for HIV-infected individuals	**Agree**<br>Disagree
15	High-risk groups should be prioritized when mpox supplies are low	**Agree**<br>Disagree

Notes: *ART means antiretroviral therapy. “Agree” as correct answer.

Table S2. Mpox-related questions and answers among male sex workers, in China.

No.	Questions	Answers
1	Ever heard of mpox:	No<br>Yes
2	Paid attention to mpox outbreak information:	Always<br>Never<br>Often
3	Got mpox information from internet:	No

		Yes
4	Got mpox information from traditional media:	No<br>Yes
5	China will become mpox endemic country:	No<br>Yes
6	Ever heard of the smallpox vaccine:	No<br>Yes
7	Ever been vaccinated against smallpox:	No<br>Unsure<br>Yes
8	Suitable for HIV-infected to get non-replicating smallpox live:	No<br>Yes
